# How to Get a Wife in India

**Published:** 1881-02

**Authors:** 


### HOW TO GET A WIFE IN INDIA.

When a man in decent rank of life wishes to marry, and can prove that he possesses the means of maintaining a wife, it is customary for him to apply to the mistress of the Byculla School, state his wishes and qualifications, and inquire into the number and character of the marriageable girls. An investigation immediately follows as to the eligibility ; and, if all promises satisfactory, he is forthwith invited to drink tea with the school-mistress upon an appointed evening, to give him an opportunity of making his selection. The elder girls are then informed of his intended visit and its purport ; and those who desire to enter the matrimonial lists come forward and signify their wish to join the party. Frequently four or five competitors make their appearance on these occasions in the mistress' room. The gentleman, while doing his best to make himself universally agreeable, yet contrives, in the course of the evening, to mark his preference for one particular lady. Should these symptoms of budding affection be favorably received, he tenders his proposal in due form on the following morning. But it often occurs that the selected lady does not participate in the inamorata's sudden flame, in which case she is at perfect liberty to decline the honor of his alliance, and reserves herself for the next tea-party exhibition.

We have known an instance when an amorous old gentleman from an out-station presented himself three successive times at these soirées, in the hope of obtaining a wife to cheer the solitude of his up-country residence, but all in vain ; the young ladies unanimously rejected him with the highest disdain, wondering "how such an ugly old fellow could have the impudence to think of a wife?"

But a different reception is given to the dashing young sergeant or smart-looking conductor; their attentions are never repulsed, and the announcement of the "chosen intendeds," as Miss Squeers would say, is anticipated with the utmost impatience by many anxious young hearts. The wedding speedily follows, the bride's modest "trousseau" being provided from the funds of the establishment, and every girl in the school cheerfully contributing her aid in the manufacture of dresses.—*Life in Bombay.*

---

"When I was your age," said old Mr. Tret, "I rose with the lark." "I beat you clear out of sight, then," said Tom, wearily and triumphantly. "I've been up all night with him."

